# Right Ventricular Septal Pacing: A Paradigm Shift

**DOI:** 10.19102/icrm.2018.090501

**Published:** 2018-05-15

**Authors:** Sarah A. Worsnick, Parikshit S. Sharma, Pugazhendhi Vijayaraman

**Affiliations:** ^1^Geisinger Heart Institute, Wilkes Barre, PA, USA; ^2^Rush University Medical Center, Chicago, IL, USA

**Keywords:** His-bundle pacing, right ventricular apical pacing, right ventricular septal pacing

## Abstract

The right ventricular (RV) apex has been considered to be the primary site for ventricular lead implantation since the original descriptions of permanent pacing. However, long-term RV apical pacing has been shown to have negative effects on ventricular function and hemodynamics as a result of ventricular dyssynchrony. Alternative sites of ventricular pacing, particularly the RV septum and His bundle, have been evaluated for patients with a need for long-term ventricular pacing. In this article, we review the available data on the use of these alternative sites for RV pacing.

## Introduction

The right ventricular (RV) apex has been considered the primary site for ventricular lead implantation since the original descriptions of permanent pacing.^1,2^ However, long-term RV apical (RVA) pacing has been shown to have negative effects on ventricular function and hemodynamics as a result of ventricular dyssynchrony.^3^ In the last decade (2000–2010), more data have emerged on the association of RVA pacing and left ventricular (LV) systolic dysfunction, heart failure (HF), ventricular remodeling, atrial fibrillation (AF), and increased mortality.^4^ Alternative sites of ventricular pacing, particularly the RV septum and His bundle, have been evaluated for use in patients with a need for long-term ventricular pacing. In this article, we review the available data on the use of these alternative sites for RV pacing.

## Right ventricular apical pacing: the old paradigm

Several studies conducted in the past 10 years have demonstrated the adverse effects of conventional RVA pacing. The Mode Selection Trial (MOST) study included a total of 2,010 patients with sick sinus syndrome who underwent dual-chamber pacemaker implants that were programmed to either a DDD or VVI mode. At three years following implantation, HF hospitalizations were found to have occurred in 12.3% of the VVI group and 10.3% of the DDD group. Of these, 50% were due to new-onset HF. Patients with a greater than 40% ventricular pacing rate in the DDD group and those with a more than 80% pacing rate in the VVI group had a twofold risk of heart failure events [DDD adjusted hazard ratio (HR): 2.60, 95% confidence interval (CI): 1.05–6.47; p < 0.05 and VVI HR: 2.50, 95% CI: 1.44–4.36; p < 0.05]. The similar increase in HF events in patients with both DDD and VVI pacing modes suggests that RVA pacing was independently associated with higher HF events, regardless of atrioventricular (AV) synchrony.^5^ A substudy of the MOST trial further demonstrated that the risk of hospitalization for HF increased by 20% for every 10% increase in RV pacing rate. Patients who had 40% ventricular pacing or more had a risk of hospitalization for HF that was 2.5 times as high as the risk among those who had pacing less than 40% of the time.^6^

The Dual-chamber and Implantable Defibrillator (DAVID) trial and the Multicenter Automated Defibrillator Implantation Trial (MADIT) II study further confirmed the relationship between RV pacing and adverse cardiovascular outcomes. The DAVID trial included 506 patients with implantable cardioverter-defibrillators (ICDs) who were randomly assigned to dual-chamber rate-responsive pacing at a rate of 70/minute (DDDR) versus ventricular backup pacing at a rate of 40/minute. Patients programmed as DDD with a backup rate of 70 beats per minute (bpm) were paced 60% of the time, whereas the patients programmed as VVI with a backup rate of 40 bpm were paced only 3% of the time. At one year of follow-up, the number of heart failure events or death was substantially higher in the patients programmed as DDD (26.7%) versus those programmed as VVI (16.1%; 95% CI: 1.06–2.44; p = 0.03).^7^

The MADIT II study enrolled 1,232 patients with ischemic cardiomyopathy.^8^ Although this study randomized patients to receive either ICD or medical therapy, the pacing parameters for the ICD patients were similar to those used in the DAVID trial (ie, DDD: 70/minute and VVI: 40/minute). Following multivariate adjustment, patients with high RV pacing burden were found to be at a significantly increased risk for new or worsened HF (HR: 1.93; p = 0.002) and in need of appropriate ICD therapy for ventricular tachycardia/ventricular fibrillation (HR: 1.50; p = 0.02). The mortality rates were similar for high and low RV pacing (13% versus 10%) at initial follow-up. During the final eight years of follow-up, however, a high RV pacing percentage was associated with a 40% increase in the risk of death in comparison with the rate associated with low RV pacing.^8,9^

A more recent retrospective analysis of 823 consecutive patients undergoing permanent pacemaker (PPM) implantation between 2000 and 2014 for complete heart block with LV ejection fraction (LVEF) > 50% was performed by Kiehl et al.^10^ Pacing-induced cardiomyopathy (PICM), defined as a cardiac resynchronization therapy (CRT) upgrade or post-PPM LVEF ≤ 40%, occurred in 101 (12.3%) patients over the mean follow-up period of 4.3 years ± 3.9 years. RV lead position was distributed relatively evenly between apical and nonapical positions. Post-PPM LVEF was 33.7% ± 7.4% in patients with PICM versus 57.6% ± 6.1% in patients without PICM (p < 0.001). In multivariable analysis, lower pre-PPM LVEF and RV pacing percentage (> 20%) were independently associated with PICM.^10^

Alternate right ventricular pacing sites

The adverse effects associated with long-term RVA pacing have led to further investigation of alternate RV pacing sites. Additional sites within the right ventricle that have been evaluated include the RV outflow tract (RVOT); RV septum (further divided into the mid- and low septum); and, more recently, the bundle of His near the AV junction. In the 1970s, Durrer et al. documented that the septal region of the RVOT and the mid-RV are the first areas within the ventricle to depolarize and, hence, pacing from these areas could theoretically recreate as normal a conduction and contraction pattern as possible.^11^ In addition to improved synchrony and conduction times, the RVOT and septal area have experienced growing popularity due to the ease of lead implantation and stability.^12,13^

**Figure 1:**
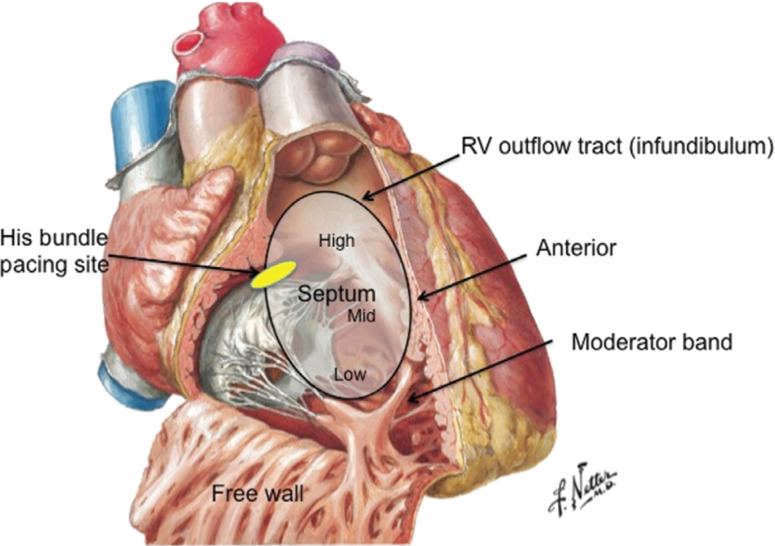
RV anatomy and a summary of RV pacing sites. Figure reproduced from the *Netter Collection of Medical Illustrations* with permission from Elsevier, Inc.

At this time, anatomy for RV pacing sites has not been standardized in the literature **(Figure 1)**. The anatomical RVOT is defined as the area between the pulmonic valve superiorly, the upper roof of the tricuspid valve apparatus inferiorly, the septum posteriorly and medially, and the free RV wall anteriorly. The proposed target zone for pacing purposes is the septoparietal trabeculations in the inferior portion of the septal RVOT, or the septal (not the free wall) zone bounded by the supraventricular crest and the septomarginal trabeculation. The zone at or below the level of the moderator band is too close to the apex and the high RVOT septum in the infundibulum/conus arteriosus is smooth-walled and demonstrates poor attachment of leads and high pacing thresholds.^14,15^

## Lead implantation

### Implantation technique

**Figure 2:**
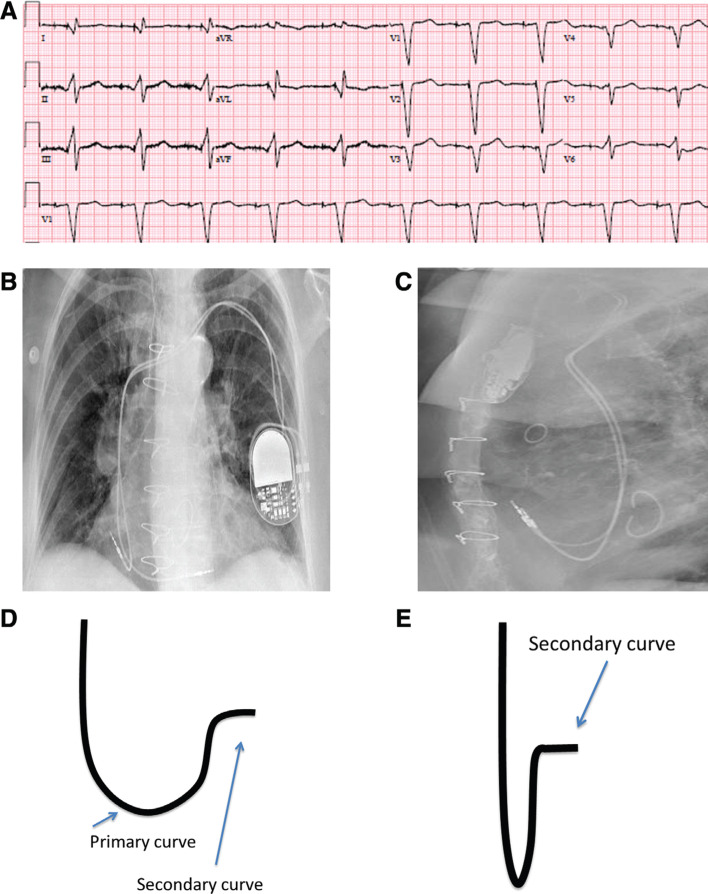
**A:** A 12-lead ECG with atrial and RV low septal pacing; QRS duration (QRSd): 150 ms. Note the Q wave in leads I and aVL. **B:** A posterioanterior view of the right atrial and septal RV leads on chest X-ray. **C:** A lateral view of the right atrial and septal RV leads. **D:** Schematic representation of the stylet shape showing the primary and secondary curves. **E:** Foreshortened view of the stylet shape demonstrating the secondary curve.

Various methods have been described for implanting pacing leads in the RVOT and RV septal region.^15–17^ Delivery of pacing leads to the appropriate location in the RVOT or RV septum from the subclavian/axillary approach via the superior vena cava requires a stylet shaped with primary and secondary curves (instead of the regular straight stylet). The primary curve is required to transverse the tricuspid valve towards the RVOT away from the apex, and a secondary posterior curve **(Figures 2D and 2E)** then redirects the lead towards the septum, away from the anterior free wall.

Any 6-French (Fr) or 7-Fr active fixation lead can be utilized for pacing. The Mond™ stylets, specifically the 4140 stylet with a medium curve and the 4150 stylet with a broader curve (Abbott Laboratories, Chicago, IL, USA), for example, have been commonly used for RVOT and septal lead placement. In general, the medium curved stylet is considered for use first, while the broader curve is reserved for employment in patients with an enlarged RV. Two stylets with differing stiffness are provided for each model. Lead manipulation is often easier with a firm stylet. Straight stylets also have the ability to be hand-shaped at the time of implant. Typically, a curve is first created at the distal 6 cm of the stylet. The terminal 2 cm is then bent to create a “swan neck deformity.”^13,16^ Lastly, while the curved end of the stylet is held with the summit superior, the terminal straight end bend is shaped toward the operator in order to ensure posterior angulation.^14^

The most common problem encountered during septal lead placement is inadvertent attachment of the lead to the anterior RV or RV free wall. When the pacing lead crosses the tricuspid valve with a simple curved stylet, it is directed superiorly towards the pulmonary valve. However, unless the top is arching posteriorly at the time of screw deployment, it will attach to the anterior or free wall. Clinical experience with the operator-curved stylet has yielded a 90% success rate with respect to RVOT septal and mid-RV septal placement of ventricular pacing leads.^18^

An alternative delivery technique using a lumenless active fixation screw in a 4.1-Fr lead (SelectSecure^®^; Medtronic Inc., Minneapolis, MN, USA) and the SelectSite™ steerable or C315 type fixed shape lead delivery system (Medtronic Inc., Minneapolis, MN, USA) is also available. In the largest published series of 138 ventricular implantations, using this system, Zanon et al. found a 98% success rate in implanting RVOT leads, with a mean fluoroscopic time of 19 minutes ± 15 minutes and only one perforation and two acute lead dislodgements, respectively.^19^ As judged by the left anterior oblique (LAO) and right anterior oblique (RAO) as well as electrocardiogram (ECG) vectors, 76% achieved a high RVOT, 6% achieved a free wall RVOT, and 18% achieved a low septal position, respectively.

Actual implants can be performed from either a left- or right-sided approach. As previously discussed, a 6-Fr or 7-Fr active fixation lead can be used, but should be at least 58 cm in length, especially in cases involving implantation from the left side. As noted, prior to insertion, a septal curve is made by the operator.

While there are several different approaches for lead insertion, the most common one is to advance the lead and stylet across the tricuspid valve and toward the RV apex. With the stylet slightly retracted, the lead can then be arched into the RVOT and the tip can subsequently prolapse into the pulmonary artery.^18,20^ In the posterior–anterior projection, the lead position can be confirmed in the pulmonary artery. Once confirmed, the lead and stylet can be retracted into the RVOT or mid-RV, in order to gain contact with the septal wall. If unsuccessful following several attempts, the steps can be repeated either with the stylet partially withdrawn or using a wider stylet model. Once the lead tip makes contact with the septal wall, the screw can be deployed and the stylet can be withdrawn. Lead placement can be confirmed best in the 40-degree (°) LAO view. The usual RV lead parameters should be tested; these include impedance, R-waves, and pacing threshold.^15^

Once testing is completed and the results are deemed satisfactory, lead slack should be left in the right atrium and RV so as to prevent lead dislodgement. This is typically performed by advancing more lead until it bends across the tricuspid annulus. Pocket closure and follow-up can be performed per the operator’s preference **(Figure 2)**.^18,20^

### Confirming lead locations

**Figure 3:**
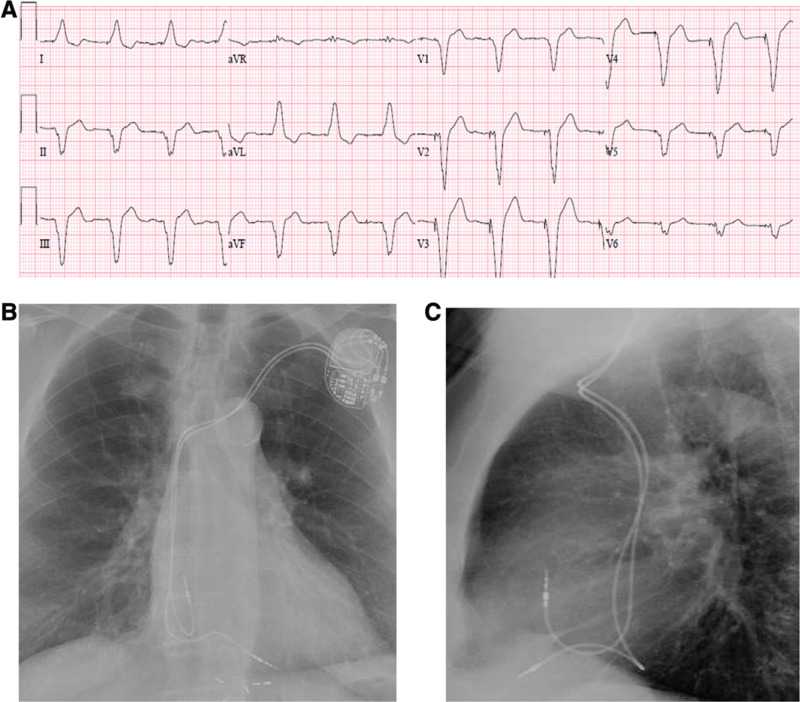
**A:** A 12-lead ECG during RV apical pacing; QRSd: 160 ms. Note the positive R-waves in leads I and aVL and the QS complexes in the chest leads. **B:** A posteroanterior chest X-ray of the right atrial and RV apical leads. **C:** A lateral chest X-ray of the right atrial and RV apical leads.

A combination of fluoroscopic images along with ECG tracings is often useful in determining and confirming lead tip positions during implantation. To help distinguish high versus low septal positions, the use of RAO fluoroscopic views is recommended, whereas the LAO 40° view is best for differentiating the RVOT septum from the free wall. Pacing morphology might also be helpful in distinguishing RV septal location from free wall locations. Pacing from the RV septum is noted by a negative QRS morphology in lead I on a 12-lead ECG, whereas pacing in the RV free wall manifests as a positive QRS morphology in lead I **(Figures 2 and 3)**. Ventricular pacing in a high-septal position will result in an upright QRS in lead aVF, whereas a lower septal position will have a less-positive QRS deflection in lead aVF.^14,15^ It is important to bear in mind that the placement of RV pacing leads using standard fluoroscopic views alone is often imprecise, and fluoroscopy, when compared with multi-slice computed tomography, is only 37%. RV leads believed to be positioned in the septum using X-ray technology were in fact very frequently placed in the RV anterior free wall.^21^

Echocardiography can be used to locate RV pacing sites and to assess the dyssynchrony associated with pacing from these sites. However, such evaluations are difficult to achieve intraprocedurally with sterility in the operating room or electrophysiology laboratory, and are more time-consuming and expensive in terms of equipment and personnel involvement.

## Studies evaluating right ventricular outflow tract and right ventricular septal pacing

As noted above, there is a lack of standardization regarding anatomical definitions of alternative pacing sites. Furthermore, the true location of the tip of the pacing lead using fluoroscopy is often inaccurate. Consequently, the resultant acute and chronic studies of RVOT and septal pacing have produced conflicting results, making interpretation of the published literature difficult. Some studies have reported that septal pacing recreates a more synchronous contraction and narrow QRS complex with preserved LV function, while others have suggested there is no benefit of septal pacing over RVA pacing in maintaining LV function **(Table 1)**.

The RV Outflow Versus Apical Pacing (ROVA) trial was a randomized crossover study aimed at determining whether quality of life (QOL) is better after three months of RVOT versus RVA pacing in 103 pacemaker recipients with HF, LV systolic dysfunction (LVEF ≤ 40%), and chronic AF. RVOT and dual-site RV pacing shortened QRS duration but, after three months, did not consistently improve QOL or other clinical outcomes as compared with RVA pacing.^22^

Zou et al. assessed RVOT septal pacing in their retrospective analysis of 80 patients with complete AV block and normal cardiac function.^2^ Patients who received either RVA pacing (n = 42) or RVOT septal pacing (n = 38) were included.^2^ During two years of follow-up, six patients developed incident AF in the RVA pacing group, while only one patient with new-onset AF was observed in the RVOT septal pacing group. Additionally, RVOT septal pacing resulted in a shorter QRS duration, intraventricular mechanical delay, and less left atrial volume increase as compared with RVA pacing. The final LVEF of the RVOT septal pacing group was also significantly higher than that of the RVA pacing group (p < 0.05).

Shimony et al. conducted a meta-analysis of randomized controlled trials comparing the mid- and long-term effects of RVA pacing and RV nonapical (RVNA) pacing.^23^ Fourteen randomized controlled trials including 754 patients from 1999 to 2010 were reviewed. It was noted that those patients randomized to RVNA pacing at 12 months’ follow-up had greater LV function (95% CI: 2.79–12.27). This was particularly noted when the LVEF was reduced at baseline (specifically < 40%–45%), or when the study duration was greater than one year. Interestingly, the higher LVEF did not correlate with improved clinical outcomes, though no published study was powered to evaluate long-term survival. Additionally, the small sample size and limited data on exercise capacity, New York Heart Association functional class, QOL, and survival yielded inconclusive results within these trials.^23^

However, the Protection of LV Function During RV Pacing (PROTECT-PACE) trial, a randomized prospective multicenter study that compared change in LVEF between RVA pacing and high-septal RV pacing over a two-year period, had contrasting findings. The primary endpoint was intrapatient change in LVEF as assessed by transthoracic echocardiography (TTE). The secondary endpoints included death or hospitalization for HF, AF burden, changes in brain natriuretic peptide (BNP) levels, six-minute walk test results, lead placement times, and lead-related adverse events. A total of 240 patients (average age: 74 years, gender: 67% male) with high-grade AV block who required > 90% ventricular pacing with a preserved LVEF were included and evenly split between the two pacing groups. Lead implantation was performed using a steerable sheath/lead system (SelectSecure^®^ Model 3830; Medtronic Inc., Minneapolis, MN, USA). In the RVA pacing group, 119 of 120 patients were successfully implanted. One hundred nine of them (92%) were classified as apical. In the RV high-septal group, 118 of 120 patients were successfully implanted. Sixty-six percent of these were deemed to be septal in nature based on radiologic imaging and review by the lead adjudication committee, despite the use of the steerable delivery system, while 30% were nonseptal and 3% had an indeterminate or inconclusive position. At two years’ follow-up, LVEF was found to be declined in both groups (RVA pacing group: 57% ± 9% to 55% ± 9%; p = 0.047 and RV high-septal pacing group: 56% ± 10% to 54% ± 10%; p = 0.0003). No significant differences were seen with respect to HF hospitalizations, AF burden, or mortality between the two groups. Additionally, no clear benefit for placing an RV high-septal lead rather than an RVA lead and vice versa in the first two years of this study was found. Notably, placement of a lead in the RV high-septal position also yielded both longer procedural and fluoroscopy times (procedure: 70 minutes ± 25 minutes versus 56 minutes ± 24 minutes; p < 0.0001 and fluoroscopy time: 11 minutes ± seven minutes versus five minutes ± four minutes; p < 0.0001).^24^

## His-bundle pacing

**Figure 4:**
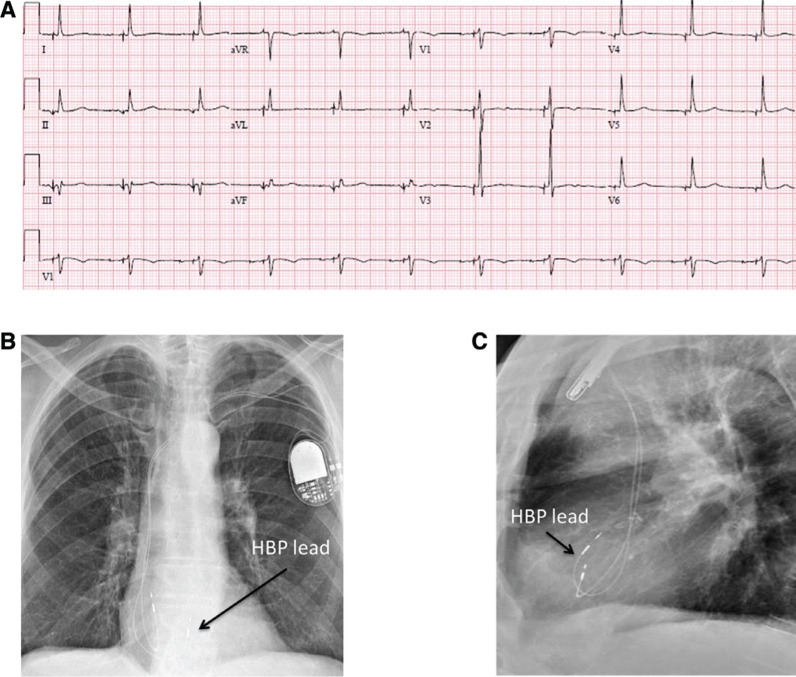
**A:** A 12-lead ECG with atrial and selective His-bundle pacing; QRSd: 90 ms. Note the normal-appearing QRS complexes with no T-wave changes. **B:** A posteroanterior chest X-ray of the right atrial and His-bundle pacing leads.** C:** A lateral chest X-ray of the right atrial and His-bundle pacing leads. HBP: His-bundle pacing.

His-bundle pacing is a physiologic form of septal pacing that has recently gained popularity due to its ability to closely reproduce native His-Purkinje-mediated ventricular activation from both electrical and hemodynamic standpoints. In permanent His-bundle pacing, the pacing lead is placed at the AV septum, targeting the main His bundle using electrogram (EGM) mapping **(Figures 1 and 4)**.

While His-bundle pacing was first described in 2000, several concerns due to reported high pacing thresholds, shorter battery life, and increased risk for lead dislodgement limited its adoption for years. The creation of dedicated pacing lead and delivery systems, however, has resulted in successful and reliable lead placement, lower thresholds, and decreased procedural times. These tools include the SelectSecure^®^ 3830 lead, which is a 4-Fr active fixation lead, and the SelectSite™ C304 and C315 delivery sheaths (Medtronic Inc., Minneapolis, MN, USA). Recent data have also shown that the location of the His bundle can be identified in 95% of patients using these tools only, without the need for a mapping catheter.^25^ Vascular access can be obtained in the usual fashion via either a cephalic, axillary, or subclavian approach. A guidewire is then advanced into the right atrium and/or RV. In our practice, this guidewire is exchanged with a SelectSite™ C315 nondeflectable delivery sheath (Medtronic Inc., Minneapolis, MN, USA). The pacing lead can then be advanced to the tip of the sheath, with the distal tip of the lead minimally exposed. The His EGMs are then mapped in a unipolar fashion from the lead tip.

If the sheath and the lead tip are in the RV, they can be gently pulled back to the AV grove with minimal counterclockwise rotation to ensure that the lead tip is abutting the septum. If the sheath and the lead are in the right atrium, gentle forward clockwise rotation helps to move the lead to the summit of the tricuspid annulus. During positioning, it is important for both the operator and the person operating the pacing system analyzer/electrophysiology recording system to closely monitor intracardiac EGMs for His-bundle signals. A sweep speed of 50 mm/s to 11 mm/s allows for better separation and identification of the atrial, His, and local ventricular EGMs.

Once His-bundle EGMs are obtained, unipolar pacing is performed starting at 5 V @ 1 ms while simultaneously assessing 12-lead QRS morphologies. Following confirmation of His-bundle capture, the lead is turned four or five times in a clockwise direction. Injury current in the His-bundle EGM can be recorded in 40% of patients and has been associated with excellent pacing thresholds. Usually, torque is built up and transmitted to the end of the lead. Once it is released, the lead may unwind one or two rotations. It is important to use the sheath to support the lead and make good contact with the septum during this time. The sheath can then be pulled back and the lead can be advanced to allow for slack. Testing is performed in both unipolar and bipolar configurations. Starting at 5 V @ 1 ms, output is decremented to assess the patient’s response to pacing. Either selective or non-selective His-bundle pacing with thresholds of less than 2 V @ 1 ms are deemed acceptable. It is essential to use 12-lead ECG monitoring to assess His-bundle capture and to differentiate from RV septal pacing. If the patient is pacemaker-dependent, lower pacing thresholds are sought before confirming the final lead position. If the operator does not deem the pacing threshold margins to be acceptable following multiple attempts, then the lead can be placed slightly more anteriorly, with larger ventricular sensing and pacing morphology consistent with septal pacing.^26^

His-bundle pacing has been shown to be effective in patients with all types of AV block, including patients with infranodal AV block and bundle branch block. A summary of various His-bundle pacing studies is shown in **Table 2**. By pacing at the site or distal to the site of the block, recruitment of the bundle branch block is feasible.^25^ Sharma et al. performed a retrospective study involving 192 patients that compared His-bundle pacing to RV pacing.^26^ They demonstrated an 80% success rate of achieving His-bundle pacing. Over a two-year period, His-bundle pacing was associated with a significant reduction in HF hospitalizations of His-bundle-paced patients in comparison with patients with RV pacing with a pacing burden of greater than 40%.^27^ More data are also emerging on the feasibility and efficacy of His-bundle pacing in patients with indications for CRT.^28,29^

Although no long-term, large randomized trials have been conducted to date, this form of pacing may be a promising representation of physiological permanent pacing.

## Conclusions

While RVA pacing has been the primary treatment for patients with bradyarrhythmias for decades, we continue to grow aware of its potential adverse effects, including LV dysfunction, HF, AF, and even death. These have been noted despite the use of AV synchronous pacing and algorithms to reduce RV pacing burden. For patients who require long-term RV pacing, alternate pacing sites should be considered. Various options, newer tools, and improved techniques are now available for alternate RV pacing site use to increase the success of implantation as well as to decrease procedural times, with the long-term goal of preserving LV function. While the superiority of RV septal pacing over RVA pacing has not been definitively established, permanent His-bundle pacing appears very promising. Long-term and large-scale trials are still needed to confirm the efficacy and safety of these methods.

## Figures and Tables

**Table 1:** Select Trials of RVA and RVNA Pacing

Author	Trial Design	RVA (n)	RVNA (n)	RVNA Site	Follow-up	Important Characteristics		Outcome(s)	
Zou et al.^2^	Retrospective/Parallel	42	38	RVOT	2 years	•	All patients: CHB with normal ejection fraction	•	RVOT septal pacing was associated with improved atrial electrical activity in patients with normal cardiac function
Stambler et al.^22^	Parallel	37	43	RVOT	3 months	•	All patients: HF, LVEF < 40%, 64% post-AVN ablation	•	Dual-site RV pacing shortens QRS duration but does not improve QOL or clinical outcomes in comparison with RVA pacing
Kaye et al.^24^	Parallel	120	120	High-septal	2 years	•	All patients: high-grade AVB, > 90% ventricular pacing, preserved LVEF	•	No difference in LVEF, mortality, HFH, or AF burden was found
Kypta et al.^29^	Parallel	45	53	Mid-septum or RVOT	3 months	•	All patients: AVB, LVEF < 40%, no HF, MI, AF	•	No difference in BNP, LVEF, or exercise capacity was found
Dabrowska-Kugacka et al.^30^	Parallel	66	56	RVOT	10 years	•	17 patients: SSS, 80 patients: AVB, 24 patients: AF, LVEF > 40%	•	No difference in all-cause or cardiovascular mortality was found
Gong et al.^31^	Parallel	44	46	RVOT	1 year	•	All patients: symptomatic AVB, LVEF > 50%	•	RVA pacing had more intra-ventricular systolic dyssynchrony than did RVNA pacing, though LVEF and left ventricular volumes were similar between the two
Cano et al.^32^	Parallel	28	32	Mid-septum	1 year	•	3 patients: SSS	•	RVA pacing had longer QRSd and more intra-ventricular dyssynchrony in comparison with RVNA pacing
						•	57 patients: AVB, LVEF > 50%	•	BNP levels, NYHA functional class, and QOL were similar between the two
Leong et al.^33^	Parallel	26	32	RVOT	11–53 months	•	26 patients: SSS	•	RVA pacing had longer QRSd and intra-ventricular dyssynchrony
						•	32 patients: symptomatic AVB	•	Left atrial volume was signiﬁcantly lower with RVNA pacing

n: number of patients; RVA: right ventricle apical; RVNA: right ventricle nonapical; RVOT: right ventricular outflow tract; CHB: complete heart block; HF: heart failure; LVEF: left ventricular ejection fraction; SSS: sick sinus syndrome; AVN: atrioventricular node; NYHA: New York Heart Association; QOL: quality of life; AVB: atrioventricular block; BNP: brain natriuretic peptide; MI: myocardial infarction; AF: atrial fibrillation; QRSd: QRS duration; HFH: heart failure hospitalizations.

**Table 2:** Outcome Studies on Permanent His-bundle Pacing

Author	Trial Design	Follow-up	Number of Patients	Success Rate	Important Characteristics	Outcome(s)	
Sharma et al.^26^	Prospective	2 years	94	80%	Indication for pacing	•	Improvement in HFH, no significant improvement in mortality or AF
Deshmukh et al.^34^	Prospective	3 years	18	66%	Chronic AF, LVEF < 40%, QRS duration <120 ms, prior AVN ablation	•	Improvement in left ventricular dimensions, NYHA FC, and LVEF
Occhetta et al.^35^	Randomized, 6-month crossover study of RVP versus HBP	1 year	16	94%	Chronic AF, prior AVN ablation	•	Improvement in NYHA FC, 6MWT, QOL, and hemodynamics
Kronborg et al.^36^	Randomized, 12-month crossover study of HBP versus RVSP	2 years	38	84%	AVB, baseline narrow QRS, LVEF > 40%	•	Improvement in LVEF; no significant improvement in NYHA FC, 6MWT, or QOL
Pastore et al.^37^	Retrospective study; 31% HBP, 29% RVSP, and 39% RVAP	1 year	477	N/A	Complete AVB, paroxysmal AF: 26% HBP and 16% RVSP/RVAP	•	HBP was associated with a lower risk of persistent/permanent AF occurrence in comparison with both RVAP and RVSP
Vijayaraman et al.^38^	Prospective case series	70 months	20	N/A	His-Purkinje conduction, QRS duration, NYHA FC, and LVEF	•	His conduction and QRS duration remained stable; LVEF and left ventricular dimensions showed non-significant improvement in long term follow-up

HFH: heart failure hospitalizations; AF: atrial fibrillation; NYHA FC: New York Heart Association functional class; LVEF: left ventricular ejection fraction; RVP: right ventricular pacing; HBP: His-bundle pacing; AVN: atrioventricular node; 6MWT: six-minute walk test; QOL: quality of life; RVSP: right ventricular septal pacing; AVB: atrioventricular block; RVAP: right ventricular apical pacing.
